# Research on the Low-Temperature Impact Toughness of a New 100-mm Ultra-Thick Offshore Steel Fabricated Using the Narrow-Gap Laser Wire Filling Welding Process

**DOI:** 10.3390/ma17061363

**Published:** 2024-03-16

**Authors:** Zhong-Lin Hou, Hai-Quan Guo, Jia-Ji Wang, Zeng-Yang Huang, Ze-An Wang, Di-Sheng Fang, Jun Qiao

**Affiliations:** 1 School of Materials Science and Metallurgy, Laser Advanced Manufacturing Technology Center, University of Science and Technology Liaoning, Anshan 114051, China; dlut_kyo@sina.com (Z.-L.H.);; 2 State Key Laboratory of Metal Material for Marine Equipment and Application, Anshan 114051, China; 3 Quzhou Academy of Metrology and Quality Inspection, Quzhou 324000, China; 4 Harbin Welding Institute Limited Company, Harbin 150028, China

**Keywords:** ultra-thick offshore steel, narrow-gap laser wire filling welding, microstructure, low-temperature impact toughness

## Abstract

Ultra-thick offshore steel, known for its high strength, high toughness, and corrosion resistance, is commonly used in marine platforms and ship components. However, when offshore steel is in service for an extended period under conditions of high pressure, extreme cold, and high-frequency impact loads, the weld joints are prone to fatigue failure or even fractures. Addressing these issues, this study designed a narrow-gap laser wire filling welding process and successfully welded a 100-mm new type of ultra-thick offshore steel. Using finite element simulation, EBSD testing, SEM analysis, and impact experiments, this study investigates the weld’s microstructure, impact toughness, and fracture mechanisms. The research found that at −80 °C, the welded joint exhibited good impact toughness (>80 J), with the impact absorption energy on the surface of the weld being 217.7 J, similar to that of the base material (225.3 J), and the fracture mechanism was primarily a ductile fracture. The impact absorption energy in the core of the weld was 103.7 J, with the fracture mechanism mainly being a brittle fracture. The EBSD results indicated that due to the influence of the welding thermal cycle and the cooling effect of the narrow-gap process, the grains gradually coarsened from the surface of the welded plate to the core of the weld, which was the main reason for the decreased impact toughness at the joint core. This study demonstrates the feasibility of using narrow-gap laser wire filling welding for 100-mm new type ultra-thick offshore steel and provides a new approach for the joining of ultra-thick steel plates.

## 1. Introduction

The new type of ultra-thick offshore steel, due to its high strength and resistance to laminar tearing, has been widely applied in fields such as marine engineering and shipbuilding [1,2,3,4]. With marine engineering continuously advancing into deep-sea and extremely cold regions, the harsh environmental conditions have increased the demand for impact toughness of offshore steel in extremely cold conditions. However, when offshore steel is used for extended periods under conditions of high pressure, extreme cold, and high-frequency impact loads, the welded joints are prone to fatigue failure or even fractures [5,6,7,8,9,10,11].

In marine engineering, the connection of thick plates is inevitable. Among the various methods of joining thick plates, welding is the most commonly used. In welding methods for marine engineering steel, argon arc welding and submerged arc welding are often employed. However, these methods require individual insulation and solidification for each layer in multi-layer and multi-pass welding, leading to low welding efficiency. With the increasing power of lasers, laser technology has begun to be applied in the field of material welding. Compared to thermal welding, laser welding has the advantage of high energy density. The ultra-high energy density allows the weld metal to melt and cool at an extremely fast rate, achieving the effect of deep penetration welding. However, due to the small size of the laser spot, it is challenging to contact both sides of the thick plate during welding. Therefore, some scholars have proposed a method of filling the weld seam with welding wire on the basis of traditional laser welding, using a smaller butt gap for thick plate welding. This process is also known as narrow-gap laser wire filling welding.

Compared to hot melt welding, the mechanical properties of weld joints obtained using narrow gap laser wire filling welding technology are superior. Ning [12] and colleagues found that welds of D406 ultra-high-strength steel using laser multi-pass narrow gap welding are more uniform and possess higher tensile strength compared to those made with traditional TIG welding. Bunaziv [13] and his team showed that, while laser-arc hybrid welding can reduce costs compared to narrow-gap laser wire filling welding, it may face issues with poor microstructure. Kaplan [14] and associates used narrow-gap laser hot-wire welding to weld 7-mm-thick steel plates, discovering that this technique not only improves laser efficiency but also enhances weld quality. Fang [15] and others used narrow-gap laser wire filling welding on TC4, finding that the welded joint’s tensile strength was slightly higher than that of the base material. Zhang [16] and team welded TA1/Q235B bimetal plates using narrow-gap laser wire filling welding, observing a significant increase in the tensile properties of the welded joints compared to the base material. Guo [17] studied welding S60 high-strength steel with laser wire filling welding, indicating that traditional TIG welding induces higher residual stress and deformation in the welded joints. Gong [18] explored the applicability of high-power laser welding technology in the production of thick plate welding for offshore wind power, finding the welded joint area to have excellent corrosion resistance and pitting resistance, superior to the base material. Zhang [19] and team researched the microstructure and properties of 40-mm-thick high-strength steel plate welds using narrow-gap laser welding, showing that the weld strength exceeded that of the base material, with good mechanical properties of the joint. Keßler [20] and others found that compared to narrow-gap TIG, Laser multi-pass narrow gap welding could reduce material consumption and increase welding speed. Furthermore, the thickness range that can be welded using narrow gap laser wire filling welding is broader compared to other processes. Elmesalamy [21] and colleagues discovered that narrow-gap laser welding can join materials much thicker than what single-pass autogenous laser welding can handle. Zhang [22] and scholars successfully welded 60-mm-thick 304 stainless steel plates using narrow-gap laser wire filling welding. Keßler [20] and others used narrow-gap laser multi-pass welding for a 72.5-mm-thick nickel-based superalloy. Not only that, Guo [23] found that ultra-narrow-gap laser multi-pass welding not only produces welds free of cracks, pores, and lack of fusion but also reduces the requirement for high-power lasers.

However, the narrow gap laser wire filling welding process is more complex compared to traditional welding methods, which also means there are more factors that can affect the quality of the weld joint. Pu [24] examined the effect of wire feed speed on narrow-gap laser wire filling welding, concluding that excessively high speeds adversely affect weld quality. Zhang [22] studied narrow-gap laser wire filling welding of thick stainless-steel plates, finding that a wider gap size reduces the range of process parameters for defect-free welds. Therefore, many scholars have conducted research on how to minimize the impact of these adverse factors on welding quality. Guo [25] used laser wire filling welding for high-strength steel, noting that reducing laser power or increasing wire feed speed can both reduce porosity in the welds. Jiang [26] and others investigated the challenge of suppressing porosity in narrow-gap laser welding of aluminum alloys AA6061 and AA2024, suggesting that stronger stirring effects are needed to eliminate bubbles.

In recent years, with the advancement of computer technology, numerical simulation has been widely applied in the field of welding, significantly improving research efficiency and precision while also optimizing welding processes and quality control. Many scholars have combined numerical simulation with experimental studies on the temperature field, deformation field, and stress field of weldments [27,28,29,30,31,32,33,34,35,36,37,38]. For instance, Liu [39] and others combined experiments and simulations to study the microstructure and residual stress of narrow-gap laser welding of TC4 titanium alloy, finding that the growth direction of crystals in different areas aligned with the temperature gradient and noted the high transverse tensile stress near the interlayers. Furthermore, Liu [40] and colleagues analyzed the deformation of extremely thick plate welds through numerical simulation and experimentation, discovering that using symmetrical U-shaped grooves and alternating welding sequences can effectively reduce post-welding deformation. These studies not only enhance the precision of predicting and controlling the performance of welded joints but also provide a basis for improvements in welding processes.

This paper uses narrow-gap laser wire filling welding technology to weld 100-mm ultra-thick offshore steel. By employing impact testing, SEM (Scanning Electron Microscopy) analysis, and EBSD (Electron Backscatter Diffraction) testing, the study investigates the impact resistance, fracture mechanisms, and microstructural evolution of the welded joints under low-temperature conditions. The goal is to obtain welded joints of ultra-thick offshore steel with excellent low-temperature impact resistance and to provide references for the improvement and optimization of the welding process for ultra-thick plates.

## 2. Experimental Materials and Methods

To ensure the weld zone’s performance is as consistent as possible with that of the base material, the type of welding wire selected for this experiment is ER55-Ni3 (State Key Laboratory of Metallic Materials for Marine Equipment and Applications, Anshan, China), with a diameter of 1.2 mm and a melting point of 1493 °C. This welding wire exhibits strong plasticity, toughness, and impact resistance, particularly demonstrating a high level of low-temperature impact toughness. The chemical composition of the welding wire and base metal are as shown in Table 1.

The narrow gap laser wire filling welding experiment equipment is an automated laser welding system produced by KUKA Automation Company in Augsburg, Germany. It utilizes a fully automated laser control device and a modular laser unit, maximizing weld seam quality. The equipment consists of the following sub-modules: a laser device, a wire feeding device, a clamping device, and a walking mechanism. The laser uses a German-made YLS-30,000 multimode fiber laser with a maximum power of 10,000 W. The wire feeder’s speed can be precisely controlled between 0.2 m/min to 22 m/min, and the wire, pre-loaded on the feeder, is delivered to the welding gun on the KUKA robotic arm through a wire tube. The walking control system is part of the KUKA robot system, which features a six-axis welding robot with flexible movement and a positioning accuracy of up to 0.05 mm.

**Figure 1:**
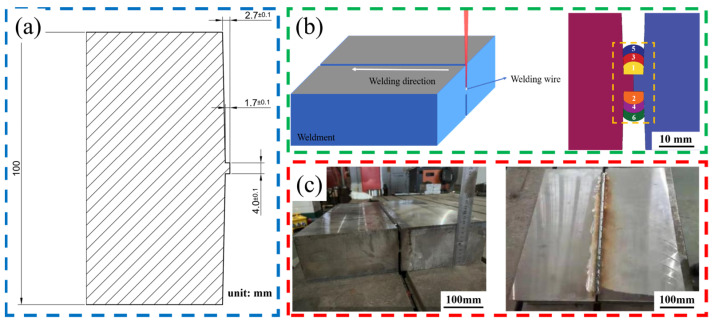
(**a**) Schematic diagram of the bevel; (**b**) Schematic of the welding process, front view of the sixth pass; (**c**) Front and top views of the finished weld.

Before welding, it is necessary to pre-treat the plates, which involves creating bevels on both the upper and lower sides. This ensures that when the two plates to be welded are joined, they form a narrow gap. The dimensions of the bevel are shown in Figure 1a. This experiment utilizes a vertical, alternating welding method. The direction of welding is consistent with the direction in which the welding wire is placed. After completing one layer of welding on one side, the base carrier platform is rotated 180° using a controller to allow for adequate cooling before welding on the other side. This process is repeated, resulting in a total of 50 layers of weld, with each layer being approximately 2 mm thick. Since air or impurities can be entrapped during welding, phenomena such as bubbles, incomplete fusion, and spatter, which affect the quality of the weld, may occur in the weld bead filling area. Therefore, it’s necessary to clean the weld bead after each layer of welding is completed. In the cleaning of the weld seam and sidewalls, an efficient and practical physical cleaning method is adopted. Specifically, we tightly wrapped coarse sandpaper around a wire, ensuring the sandpaper’s length exceeded that of the weld seam. Then, two operators, standing on either side of the workpiece, pulled the wire in coordination. This mechanical abrasion effectively removed welding spatter, bumps, and other impurities, making the weld seam surface smooth and even. After the initial sanding, we wrapped cotton soaked in alcohol around the same wire and repeated the process. This step aimed to remove oil and other subtle impurities from the weld seam surface. Once the alcohol evaporated and the surface showed no obvious oily sheen, the cleaning of the weld seam was complete, readying it for the next welding pass. The welding schematic is shown in Figure 1b, and the finished welding product is displayed in Figure 1c. Detailed welding parameters are shown in the Table S1 (Supplementary Materials).

To test the impact toughness of 100-mm-thick offshore engineering steel after welding under low-temperature conditions, this study involved cutting standard weld impact specimens from the base material, the core of the weldment, and the surface layer of the weldment. These specimens were then subjected to Charpy impact tests. All specimens conformed to the latest Chinese national standard, GB/T 229-2020, with standard dimensions of 55 mm × 10 mm × 10 mm. A single-sided V-notch with an angle of 45° and a root radius of 0.25 mm was used. The specimen was cut perpendicular to the direction of the weld. The fracture surfaces of these samples were observed at 500× and 1000× magnifications using a German-made Carl Zeiss SIGMAHD (Jena, Germany) field emission high-resolution scanning electron microscope equipped with a backscattered electron detector. This observation was conducted to study the fracture morphology and investigate the fracture mechanism. Samples were taken from the base material, the surface of the weld, at 1/4th position, and the core, with a thickness of 1 mm. These were water-ground with sandpaper of varying grit sizes until the observation surfaces were smooth and flat. Then, the samples were mechanically polished using 1.0 µm diamond paste. This was followed by electropolishing to eliminate the stress layer on the surface. Backscattered Electron Diffraction (EBSD) technique was used to observe the microstructure of these samples. The EBSD scanning step size was 0.75 µm, with a collection rate of 194.92 Hz. The equivalent circle diameter method was employed to calculate the grain size of the samples, and the formula for the equivalent circle diameter method is as follows [41,42,43].

(1) $$S=2\sqrt{\frac{A}{\pi }}$$

In the formula, *S* represents the average size of the grains in the sample; *A* is the area of the grains in the sample.

## 3. Finite Element Simulation Analysis

Laser welding of thick plates in offshore steel, with narrow gaps, is challenging, and due to the significant thickness, requires multiple layers and passes to complete the filling of the entire weld seam. In this welding process, the wire melts and solidifies in the gap, forming a complex transient temperature field. During the laser welding process, the plates to be welded are heated locally and partially melted by the laser heat source. Due to current technological and cost limitations, it is difficult to experimentally measure the temperature field of the welding pool. However, welding numerical simulation technology can predict the feasibility of welding parameters to a certain extent. By simulating the temperature field of steel plate welding, the plate temperature during the welding process can be theoretically calculated, thus obtaining the temperature field and the theoretical welding pool area. By comparing the simulated weld pool morphology with the actual weld pool, the accuracy of the heat source welding model can be verified.

### 3.1. Establishment of the Model and Division of the Mesh

**Figure 2:**
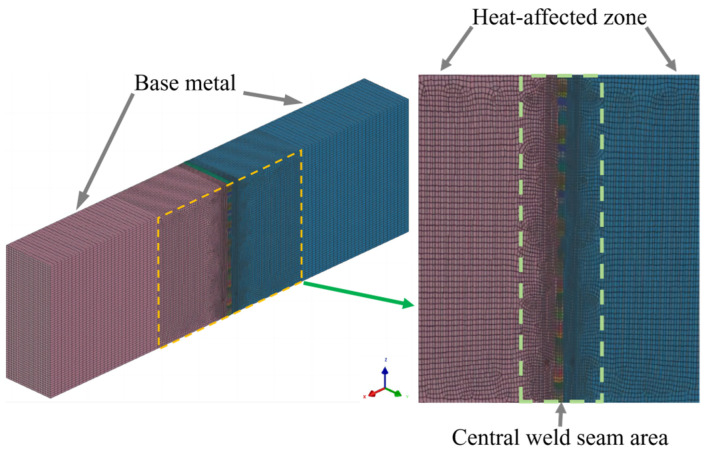
Mesh distribution of the ultra-thick plate model.

To predict temperature field changes during the welding process, finite element welding simulation software was employed to simulate the temperature field in multi-layer, multi-pass welding of laser narrow-gap thick plates. Taking into account the computational limitations, the model was simplified by reducing its length from 500 mm to 50 mm for modeling and calculation, and it comprised a total of 50 welded layers. The mesh was designed with finer granularity in the central weld seam area, measuring 0.5 mm; the heat-affected zone had a mesh size of 1 mm; and for the base material area, which is less affected by heat and farther from the heat source, the mesh size was set to 2 mm. The size and number of the grid have a significant impact on the computational results. The smaller and more densely packed the grid, and the greater its quantity, the longer the computation time required. However, this also results in higher accuracy and smaller errors in the computational outcomes. Therefore, the grid is noticeably denser in the weld bead and the heat-affected zone, and distinctly sparser towards the edges of the plate material. Details of the specific mesh division are illustrated in Figure 2.

### 3.2. Setting of Boundary Conditions

The finite element analysis software comes with three commonly used heat source models: the Gaussian plane heat source model, the Gaussian volumetric heat source model, and the double-ellipsoidal heat source model. Since this experiment involves the welding of thick plates of offshore steel, the laser heat source in the laser welding process has real-time transient characteristics and locally concentrates energy and heat, which can lead to significant changes in temperature gradients. Therefore, selecting the correct heat source welding model during the welding process plays a significant role in simulating welding results and optimizing the welding process.

Since the welding heat source is a laser beam, the Gaussian volumetric heat source model was selected. This model essentially consists of a series of planar Gaussian heat sources superimposed along the thickness direction of the workpiece. The heat flux density remains constant along the z-axis (the vertical centerline of the weld), while the radius of heat flow distribution on each cross-section linearly decreases along the thickness direction. The formula for the heat flow is as follows [44,45,46].

(2) $$Q\left(x,y,z\right)=Q_{0}\exp\left[-\frac{x^{2}+y^{2}}{r_{0}^{2}\left(z_{0}\right)}\right]$$

(3) $$r_{0}\left(z\right)=r_{e}+\frac{r_{i}-r_{e}}{z_{i}-z_{e}}\left(z-z_{e}\right)$$

In the above equation, $z_{e}$ and $z_{i}$ represent the z-axis coordinates of the upper and lower surfaces, respectively, while $r_{e}$ and $r_{i}$ denote the radii of the heat flow distribution on the upper and lower surfaces of the workpiece, as affected by the heat source.

(4) $$q\left(x,y,z,t\right)=\lambda \frac{\partial t}{\partial x}n_{x}+\lambda \frac{\partial t}{\partial y}n_{y}+\lambda \frac{\partial t}{\partial z}n_{z}$$

*λ* Is the thermal conductivity (J·mm^−1^·s^−1^·K^−1^).

Due to significant temperature gradients in the molten pool area, the heat-affected zone, the base material area, and compared to room temperature, there exists both convective and radiative heat transfer within the workpiece, as well as between the workpiece surface and the air during the welding process. In the numerical simulation of temperatures during welding, the heat exchange and cooling boundary conditions are the primary considerations. The convective heat transfer, influenced by temperature gradients and the inert gas acting on the plate, follows Newton’s law of cooling [47]. The heat bodies generate radiative heat transfer by absorbing laser light, which follows the Stefan-Boltzmann law [47].

Convective cooling:

(5) $$q_{c}^{*}=\alpha_{c}\left(T-T_{0}\right)$$

In Equation (5), $\alpha_{c}$ represents the convective surface heat transfer coefficient (J·mm^−2^·s^−1^·K^−1^). T is the temperature of the welded component (K). $T_{0}$ is the initial temperature (K).

Radiative cooling:

(6) $$q_{\gamma }^{*}=\varepsilon C_{0}\left(T^{4}-T_{0}^{4}\right)$$

In Equation (6), $C_{0}$ equals 5.67 × 10^−14^ J·mm^−2^·s^−1^·K^−4^, and *ε* represents the emissivity coefficient (absorptivity), with *ε* being less than 1.

This study employs natural air cooling for the welded components, necessitating consideration of both radiative and convective cooling. Therefore, it is essential to consider a comprehensive cooling coefficient, as shown in Equation (7) [48,49].

(7) $$\partial =\frac{q_{c}^{*}+q_{r}^{*}}{T-T_{0}}=\varepsilon C_{0}\left(T+T_{0}\right)\left(T^{2}+T_{0}^{2}\right)+\partial_{c}$$

### 3.3. Material Thermophysical Property Parameters

**Figure 3:**
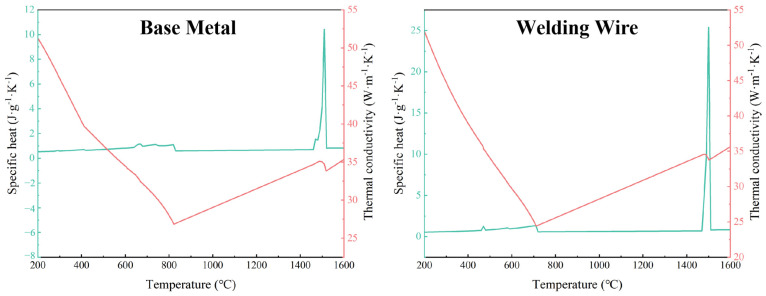
Specific heat capacity and thermal conductivity of the base metal and welding wire.

During the process of laser wire filling welding, as the laser heat source melts the wire and fills the weld bead, there is a rapid increase and decrease in the temperature on the welded part. The thermophysical property parameters of the welding plate material also change with the temperature. The variation in these parameters affects the computational accuracy of finite element simulation to varying degrees and also impacts the changes in the morphology of the weld pool during the welding process. To ensure the accuracy of the computational results, it is essential to consider the changes in the material’s thermophysical property parameters. Among these, the material thermophysical parameters with the most significant impact on accuracy are specific heat capacity and thermal conductivity. In this paper, the thermophysical property parameters used in the simulation are calculated using Jmatpro 7.0 software and are set in Comsol using a piecewise linear method. Figure 3 shows the variation of welding wire and base metal thermophysical parameters with temperature.

### 3.4. Verification of the Finite Element Model

Since the results calculated by the model are based on ideal conditions, various natural environmental factors present during the experimental process may cause deviations in the experimental results. To verify the accuracy of the finite element simulation, the nodal temperatures of a five-layer filler wire welding model were compared with the real-time temperatures of the corresponding test plate material during welding. In this work, the initial temperature of the heat source welding model is set to be the same as the ambient temperature, which is 20 °C. Additionally, the laser power is set to 4500 W, with a welding speed of 0.6 m/min. 

**Figure 4:**
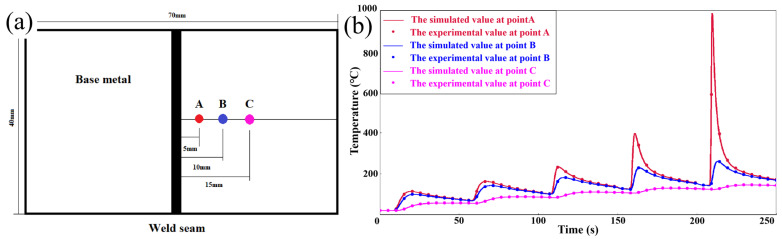
(**a**) Diagram of experimental temperature measurement locations; (**b**) Error chart of simulated and experimental thermal cycle curves.

Figure 4a shows the locations where temperatures were measured, and Figure 4b presents a comparison of the nodal temperature thermal cycling curves with the actual measured temperature curves at corresponding locations on the plate material, showing that the maximum error between the experiment and the simulation measurements was approximately 15 °C. 

**Figure 5:**
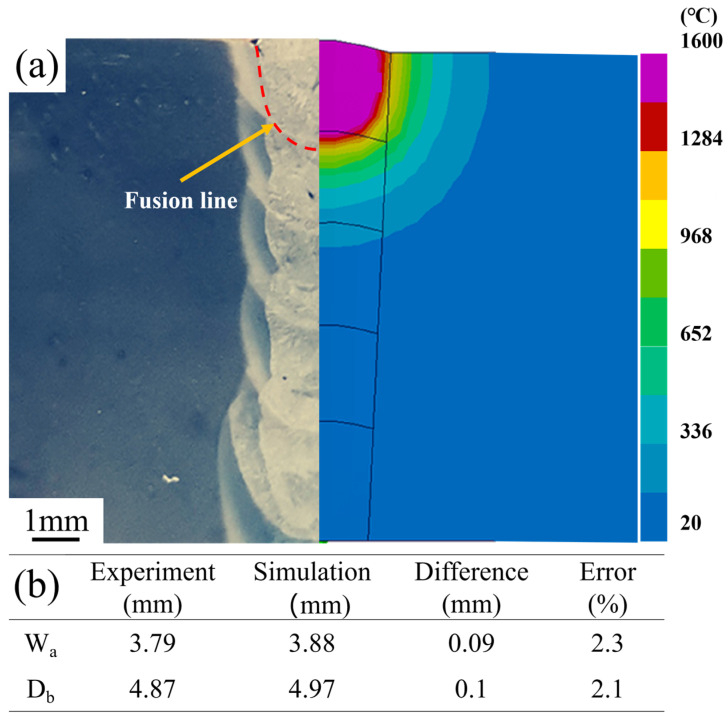
(**a**) Fitting diagram of the model and experimental weld pool morphology; (**b**) Simulation and experimental results for weld depth and width.

Additionally, as shown in Figure 5a, a comparison of the model’s molten pool cross-sectional image with the actual cross-sectional image of the plate material was made. The simulated molten pool morphology closely matches the actual fusion line of the weld. Figure 5b lists the values of weld depth (*D_b_*) and weld width (*W_a_*) obtained through experimental measurement and simulation calculation. The errors in simulated weld width and depth compared to the actual measurements were 2.3% and 2.1%, respectively. These results collectively indicate that the computational model possesses a certain degree of accuracy.

### 3.5. Finite Element Temperature Field Simulation Analysis

**Figure 6:**
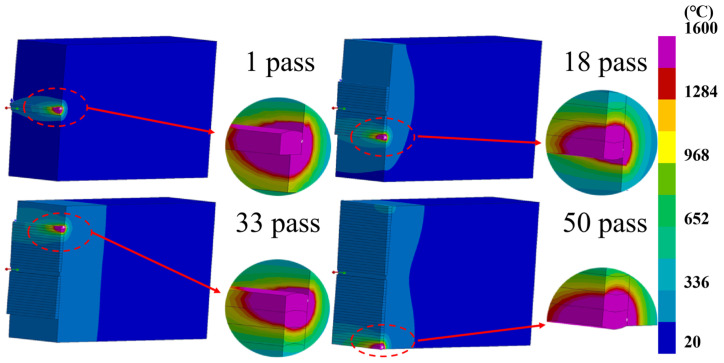
Temperature contour maps for the 1st, 18th, 33rd, and 50th passes.

Figure 6 displays the temperature field distribution cloud maps of the 1st, 18th, 33rd, and 50th layers of the welded plate at the moments of heat source application. It is evident from the first pass in the figure that the temperature influence is confined to the weld seam area and the heat-affected zone. As the welding progresses through the 18th, 33rd, and 50th passes, the range of temperature influence gradually expands until it spreads across the entire surface. During the welding process, due to the influence of the welding thermal cycle and the constraints of narrow-gap cooling, the core of the weldment maintains a relatively high temperature for an extended period. This could lead to some differences in the microstructure between the core and the surface of the plate.

## 4. Results and Discussion

### 4.1. Impact Toughness Test

**Figure 7:**
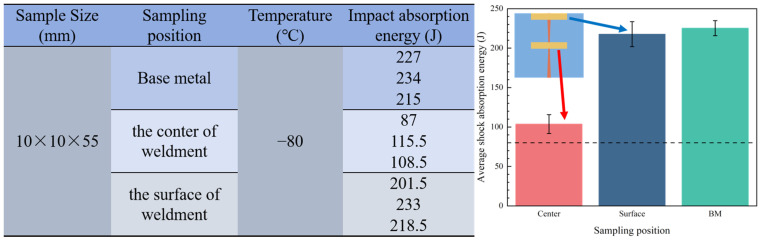
Results of the impact test at −80 °C.

To investigate the low-temperature impact toughness of the welded joints, this study conducted impact tests on samples from different locations at −80 °C. The locations of the impact test samples and the experimental results are shown in Figure 7. As can be seen from the figure, the impact absorption energy of the base material and the weldment surface is close, at 225.3 J and 217.7 J, respectively. The impact energy of the core of the weldment is 103.6 J, which is 52.45% lower than that of the weldment surface, but still higher than the allowable impact energy (80 J) at −80 °C conditions.

**Figure 8:**
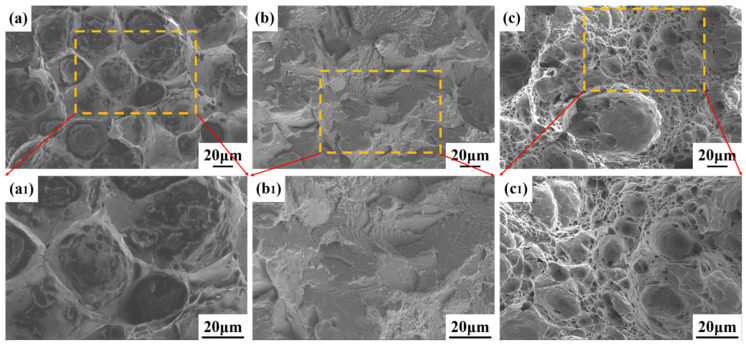
Fracture morphology of impact specimens. (**a**,**a1**) Fracture morphology of the base material; (**b**,**b1**) Fracture morphology of the central part of the weldment; (**c**,**c1**) Fracture morphology of the surface of the weldment.

To investigate the fracture mechanism of the impact specimens, this study used a Scanning Electron Microscope (SEM) to analyze the fracture surfaces. Figure 8a–c shows the fracture morphologies of the base material, the core of the weldment, and the surface of the weldment, respectively. The results in Figure 8a,c reveal the presence of dimples of varying sizes, indicating that the fracture mechanisms in both the base material and the weldment surface are primarily ductile. However, the fracture morphology of the weldment core (as shown in Figure 8b) displays a distinct river pattern, indicating that the core of the weldment experienced brittle fracture, hence the impact toughness of this area is lower compared to that of the base material.

### 4.2. Microstructural Analysis

**Figure 9:**
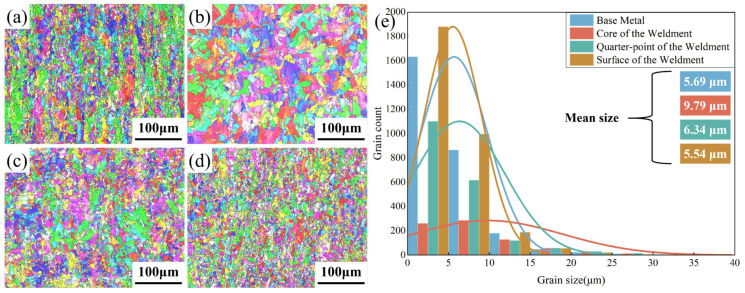
(**a**) EBSD result diagram of the base material; (**b**) EBSD result diagram of the central part of the weldment; (**c**) EBSD result diagram at 1/4 distance into the weldment; (**d**) EBSD result diagram of the surface of the weldment; (**e**) Distribution and average size of the grains in the sample.

To explore the relationship between the microstructure of welded samples and their impact toughness, this study used Electron Backscatter Diffraction (EBSD) to analyze the microstructures of the base material, the core of the weldment, the quarter point of the weldment, and the surface of the weldment. The sampling locations and experimental results are shown in Figure 9. The results indicate that the average grain size of the base material is 5.69 µm. In the welded plate, the grain size gradually increases from the surface layer to the core of the weld joint, growing from 5.54 µm to 9.79 µm, an increase of 76.71%.

Combined with the welding process and the temperature distribution cloud map, it is evident that the laser heat source repeatedly acts on the weld seam, causing multiple cycles of heating in the core of the weldment. Additionally, the heat dissipation limitation of the narrow-gap process means that the core of the weldment cannot rapidly cool down. This continuous thermal cycling and heat dissipation constraint due to the narrow gap keep the core of the weldment at a higher temperature for an extended period, ultimately leading to grain coarsening in the core.

According to the grain boundary damping effect [50,51], when materials are subjected to impact or stress loading, the relative displacement of grain boundaries leads to energy dissipation, thereby slowing down the propagation speed of shock waves and energy transfer, and enhancing the material’s resistance to impact. Therefore, the finer the grain size of the material, the higher the content of grain boundaries per unit volume, and the better its resistance to impact. Compared to the surface layer of the weldment, the core has larger grains, resulting in a reduced content of grain boundaries per unit volume and a weakened grain boundary damping effect. When subjected to impact, the core of the weldment cannot effectively absorb or dissipate the impact energy. Instead, the energy internally accumulates and concentrates between the grains, leading to stress concentration in the core of the weldment and ultimately reducing the impact toughness of the samples in that area.

## 5. Conclusions

This study thoroughly explored the feasibility of narrow-gap laser filler wire welding in the joining of 100 mm new type extra-thick offshore steel. It employed a comprehensive approach using finite element simulation, Electron Backscatter Diffraction (EBSD) analysis, Scanning Electron Microscopy (SEM) inspections, and impact tests to investigate the microstructure, impact toughness, and fracture mechanisms of the weldments.

Through the designed welding process parameters and strict experimental operations, we successfully completed the welding of 100 mm new type extra-thick offshore steel using narrow-gap laser filler wire welding technology. In subsequent performance tests, the weldment displayed excellent impact toughness at −80 °C, with an impact absorption energy exceeding 80 J, fully demonstrating the application potential of narrow-gap laser filler wire welding in the joining of extra-thick offshore steel.

Further testing of the impact performance in different parts of the weldment revealed that the impact absorption energy on the weldment’s surface reached 217.7 J, close to the base material’s 225.3 J, indicating that the mechanical properties of the weld joint’s surface were well-preserved. Meanwhile, the impact absorption energy at the core of the weldment was 103.7 J, showing a decrease compared to the surface. Analysis of the fracture mechanisms revealed that the fracture of the weldment’s surface was primarily ductile, while the core exhibited certain brittle fracture characteristics.

To delve into the reasons behind the decrease in impact toughness at the core of the welded samples, we conducted a detailed analysis of the microstructure. The results indicated that under the combined effects of the welding thermal cycle and the narrow-gap process’s heat dissipation, the grain size gradually increased from the sample’s surface to its core, from 5.54 µm to 9.79 µm, an increase of 76.71%. This grain coarsening phenomenon is the main reason for the decrease in impact toughness at the core of the welded samples.

This research demonstrates the feasibility of narrow-gap laser filler wire welding in the joining of extra-thick offshore steel. These findings not only showcase the technical advantages of narrow-gap laser filler wire welding in the welding of extra-thick offshore steel but also provide valuable references for the improvement of thick plate joining processes and the optimization of welding performance.

## Figures and Tables

**Table 1:** Chemical composition of the welding wire and base metal.

Element	C	Si	Mn	Cu	Ni	Cr	Mo	Nb	V	Ti	P	S
base metal	0.06	0.2	1.44	0.25	0.58	0.21	0.22	0.03	0.04	0.011	-	-
welding wire	0.1	0.6	3.35	-	3.35	-	-	-	-	-	0.01	0.008

## Data Availability

Data are contained within the article.

## Supplementary Materials

The following supporting information can be downloaded at: https://www.mdpi.com/article/10.3390/ma17061363/s1, Table S1: Welding parameters.
