# Supplementary material for: Research on the Low-Temperature Impact Toughness of a New 100-mm Ultra-Thick Offshore Steel Fabricated Using the Narrow-Gap Laser Wire Filling Welding Process
Source: Materials (Basel). 2024 Mar 16;17(6):1363. doi: 10.3390/ma17061363 (PMC10972308; doi:10.3390/ma17061363)
Supplement: Supplementary file 1 [file materials-17-01363-s001.zip › materials-2865465-supplementary.pdf]

Table S1. Welding parameters

Layers	Laser Power (kW)	Welding Speed (mm/min)	Wire Feed Speed (m/min)	Defocus Amount (mm)	Wobble Frequency (Hz)	Wobble Amplitude (mm)
1-1	3000	300	0	+15	200	3
2-1	3000	300	0	+15	200	3
1-2	3300	300	3	+15	200	3.2
2-2	3300	300	3	+15	200	3.2
1-3	3300	300	3	+15	200	3.2
2-3	3300	300	3	+15	200	3.2
1-4	3300	300	3	+15	200	3.2
2-4	3300	300	3	+15	200	3.2
1-5	3300	300	3	+15	200	3.5
2-5	3300	300	3	+15	200	3.5
1-6	3300	300	3	+15	200	3.5
2-6	3300	300	3	+15	200	3.5
1-7	3300	300	3	+15	200	3.5
2-7	3300	300	3	+15	200	3.5
1-8	3300	300	3	+15	200	3.5
2-8	3300	300	3	+15	200	3.5
1-9	3300	300	3	+15	200	3.5
2-9	3300	300	3	+15	200	3.5
1-10	3500	300	3	+15	200	3.8
2-10	3500	300	3	+15	200	3.8
1-11	3500	300	3	+15	200	3.8
2-11	3500	300	3	+15	200	3.8
1-12	3500	300	3	+15	200	3.8
2-12	3500	300	3	+15	200	3.8
1-13	3500	300	3	+15	200	3.8
2-13	3500	300	3	+15	200	3.8
1-14	3500	300	3	+15	200	4.0
2-14	3500	300	3	+15	200	4.0
1-15	3500	300	3	+15	200	4.0
2-15	3500	300	3	+15	200	4.0
1-16	4000	300	3	+15	200	4.0
2-16	4000	300	3	+15	200	4.0
1-17	4000	300	3	+15	200	4.0
2-17	4000	300	3	+15	200	4.0
1-18	4000	300	3	+15	200	4.0
2-18	4000	300	3	+15	200	4.0
1-19	4000	300	3	+15	200	4.2
2-19	4000	300	3	+15	200	4.2
1-20	4000	300	3	+15	200	4.2
2-20	4000	300	3	+15	200	4.2

Layers	Laser Power (kW)	Welding Speed (mm/min)	Wire Feed Speed (m/min)	Defocus Amount (mm)	Wobble Frequency (Hz)	Wobble Amplitude (mm)
1-21	4300	300	3	+15	200	4.2
2-21	4300	300	3	+15	200	4.2
1-22	4300	300	3	+15	200	4.2
2-22	4300	300	3	+15	200	4.2
1-23	4300	300	3	+15	200	4.4
2-23	4300	300	3	+15	200	4.4
1-24	4500	300	3	+15	200	4.4
2-24	4500	300	3	+15	200	4.4
1-25	4500	300	3	+15	200	4.6
2-25	4500	300	3	+15	200	4.6
